# Progressive impairment of Ca_V_1.1 function in the skeletal muscle of mice expressing a mutant type 1 Cu/Zn superoxide dismutase (G93A) linked to amyotrophic lateral sclerosis

**DOI:** 10.1186/s13395-016-0094-6

**Published:** 2016-06-23

**Authors:** Donald Beqollari, Christin F. Romberg, Gabriella Dobrowolny, Martina Martini, Andrew A. Voss, Antonio Musarò, Roger A. Bannister

**Affiliations:** Department of Medicine-Cardiology Division, University of Colorado School of Medicine, 12700 East 19th Avenue, B-139, Aurora, CO 80045 USA; Institute Pasteur Cenci-Bolognetti, DAHFMO-Unit of Histology and Medical Embryology, La Sapienza University, Via A. Scarpa, 14, 00161 Rome, Italy; Center for Life Nano Science@Sapienza, Istituto Italiano di Tecnologia, Rome, Italy; Department of Biological Sciences, College of Science and Mathematics, Wright State University, 235A Biological Sciences, 3640 Colonel Glenn Highway, Dayton, OH 45435 USA

**Keywords:** Amyotrophic lateral sclerosis, ALS, Ca_V_1.1, Excitation-contraction coupling, L-type, Skeletal muscle, SOD1, Neuromuscular disease

## Abstract

**Background:**

Amyotrophic lateral sclerosis (ALS) is an adult-onset neurodegenerative disorder that is typically fatal within 3–5 years of diagnosis. While motoneuron death is the defining characteristic of ALS, the events that underlie its pathology are not restricted to the nervous system. In this regard, ALS muscle atrophies and weakens significantly before presentation of neurological symptoms. Since the skeletal muscle L-type Ca^2+^ channel (Ca_V_1.1) is a key regulator of both mass and force, we investigated whether Ca_V_1.1 function is impaired in the muscle of two distinct mouse models carrying an ALS-linked mutation.

**Methods:**

We recorded L-type currents, charge movements, and myoplasmic Ca^2+^ transients from dissociated flexor digitorum brevis (FDB) fibers to assess Ca_V_1.1 function in two mouse models expressing a type 1 Cu/Zn superoxide dismutase mutant (SOD1^G93A^).

**Results:**

In FDB fibers obtained from “symptomatic” global SOD1^G93A^ mice, we observed a substantial reduction of SR Ca^2+^ release in response to depolarization relative to fibers harvested from age-matched control mice. L-type current and charge movement were both reduced by ~40 % in symptomatic SOD1^G93A^ fibers when compared to control fibers. Ca^2+^ transients were not significantly reduced in similar experiments performed with FDB fibers obtained from “early-symptomatic” SOD1^G93A^ mice, but L-type current and charge movement were decreased (~30 and ~20 %, respectively). Reductions in SR Ca^2+^ release (~35 %), L-type current (~20 %), and charge movement (~15 %) were also observed in fibers obtained from another model where SOD1^G93A^ expression was restricted to skeletal muscle.

**Conclusions:**

We report reductions in EC coupling, L-type current density, and charge movement in FDB fibers obtained from symptomatic global SOD1^G93A^ mice. Experiments performed with FDB fibers obtained from early-symptomatic SOD1^G93A^ and skeletal muscle autonomous MLC/SOD1^G93A^ mice support the idea that events occurring locally in the skeletal muscle contribute to the impairment of Ca_V_1.1 function in ALS muscle independently of innervation status.

**Electronic supplementary material:**

The online version of this article (doi:10.1186/s13395-016-0094-6) contains supplementary material, which is available to authorized users.

## Background

Amyotrophic lateral sclerosis (ALS), known commonly as Lou Gehrig’s disease in the USA or motoneurone disease in the UK, is an adult-onset neurodegenerative disorder that is usually lethal within 3 to 5 years of initial diagnosis [1, 2]. As a consequence of disrupted nerve-muscle communication, afflicted individuals progressively lose control of voluntary muscle function and die most frequently from respiratory failure. Nearly a quarter of familial ALS cases have been linked to point mutations in the type 1 Cu/Zn superoxide dismutase (SOD1) enzyme, which functions to mitigate cellular oxidative stress [3]. Since these mutations are autosomal dominant, transgenic mice overexpressing ALS-linked SOD1 mutations have proven to be useful models for investigation of ALS pathogenesis [4, 5].

Two independent groups have found that skeletal muscle-targeted, transgenic overexpression of SOD1^G93A^ causes profound muscle atrophy [6, 7] and initiates motoneuron death [7]. These congruent reports reinforce earlier challenges to the dogma that ALS is solely a neurological disorder and support the “dying-back” phenomenon by which motor unit loss and associated muscle function precede the death of motor neurons (reviewed in refs. [8–11]). Additional support for this view is provided by the observations that destruction of neuromuscular junctions has been linked to oxidative stress induced by tissue-specific breakdown of muscle mitochondria [12] and trophic factors secreted from the skeletal muscle promote motoneuron survival in an ALS model by stabilizing neuromuscular junctions (e.g., IGF-1, GDNF) [13–15].

In the skeletal muscle, Ca_V_1.1 functions both as the voltage-sensor for excitation-contraction (EC) coupling and as an L-type Ca^2+^ channel [16–18]. Ca_V_1.1’s role as voltage-sensor is firmly established [19], but recent work has also revealed that L-type Ca^2+^ entry maintains myoplasmic Ca^2+^ levels during repetitive activity [20, 21] augments muscle contraction [22], engages excitation-transcription coupling [23], regulates energy expenditure [24], and promotes development of neuromuscular junctions [25]. Thus, aberrant Ca_V_1.1 activity may profoundly affect skeletal muscle biology.

The question of whether Ca_V_1.1 function is disrupted during the progression of ALS was originally posed over 20 years ago [26–28], but the experimental approach used to investigate this premise left much ambiguity. In these earlier studies, serum of ALS patients was acutely applied to normal cut wild-type rat extensor digitorum longus (EDL) fibers and the Vaseline-gap voltage-clamp technique was used to record L-type currents and gating charge movements [26, 27]; L-type currents and intramembrane gating charge movements were both found to be reduced. Similarly, acute IgG application also produced inhibitory effects on single Ca_V_1.1 channels in planar lipid bilayers [28]. In the time that has passed since the experiments that employed acute application of ALS IgG were performed, it has become evident that that muscle dysfunction in ALS manifests early and slowly over time. Fortunately, a number of ALS mouse models have been developed since then, including SOD1^G93A^ transgenic lines [4, 29–31].

Here, we provide the first comprehensive voltage-clamp assessment of Ca_V_1.1 function in the muscle of an engineered mammalian model of ALS. Specifically, we utilized both global SOD1^G93A^ mice and mice expressing SOD1^G93A^ only in skeletal muscle to systematically investigate whether Ca_V_1.1 function is compromised during the progression of the disease. We found substantial reductions in myoplasmic Ca^2+^ transient amplitude, peak L-type current density, and maximal intramembrane charge movement in flexor digitorum brevis (FDB) fibers obtained from “symptomatic” congenic SOD1^G93A^ mice. Consistent with a progressive muscle defect, we observed lesser reductions in L-type current amplitude and charge movement in FDB fibers obtained from “early-symptomatic” SOD1^G93A^ mice in which widespread denervation had yet to occur. To investigate the possibility that events occurring in muscle contribute, at least in part, to the impairment of Ca_V_1.1 function, we performed voltage-clamp experiments with FDB fibers of mice expressing SOD1^G93A^ autonomously in skeletal muscle. In these experiments, we observed significant reductions in EC coupling, charge movement, and peak L-type current amplitude.

## Methods

### Ethical information/animals

All procedures involving mice were approved by the University of Colorado-Anschutz Medical Campus Institutional Animal Care and Use Committee (91813(05)1D). Male congenic SOD1^G93A^ mice (B6.Cg-Tg(SOD1*G93A)1Gur/J; 32 transgene copies) and age-matched male C57BL/6J mice (both Jackson Laboratories, Bar Harbor, ME) were used at two time points based on the criteria for disease progression described by Hatzipetros et al. [31]: (1) “symptomatic” and (2) “early-symptomatic.” Nine symptomatic mice presented classical ALS symptoms (i.e., tremor, abnormal gait, comprised ability to stand on hindquarters, etc.) when they were sacrificed at 149 ± 1 days. Six mice from the early-symptomatic cohort were largely devoid of overt symptoms when they were sacrificed at 105 ± 1 days. Age-matched C57BL/6J mice were used as background wild-type controls for both symptomatic and early-symptomatic global SOD1^G93A^ mice (eight and seven mice, respectively). Seven MLC/SOD1^G93A^ mice were sacrificed at (111 ± 1 days) [6]. In this case, six age-matched FVB/NJ mice were used as the background wild-type control strain. The dissimilar backgrounds precluded direct comparisons between global SOD1^G93A^ and MLC/SOD1^G93A^ strains.

### Dissociation of FDB fibers

The FDB muscles were dissected in cold (~4 °C) Rodent Ringer’s solution (in mM: 146 NaCl, 5 KCl, 2 CaCl_2_, 1 MgCl_2_, 10 HEPES, pH 7.4 with NaOH). Muscles were then digested in a mild collagenase solution (155 Cs-aspartate, 10 HEPES, 5 MgCl_2_, pH 7.4 with CsOH, supplemented with 1 mg/ml BSA, and 1 mg/ml collagenase type IA (Sigma-Aldrich, Saint Louis, MO) with agitation at 37 °C for ~1 h. Following digestion, the collagenase solution was replaced with dissociation solution (140 Cs-aspartate, 10 Cs_2_EGTA, 10 HEPES, 5 MgCl_2_, pH 7.4 with CsOH, supplemented with 1 mg/ml BSA) and muscles were triturated gently with a series of fire-polished glass Pasteur pipettes of descending bore. Dissociated FDB fibers destined for electrophysiological experiments were then plated onto ECL (Millipore, Billerica, MA)-coated 35-mm plastic culture dishes (Falcon, Tewksbury, MA). For Ca^2+^ imaging experiments, FDB fibers were plated onto laminin (Invitrogen, Eugene, OR)-coated 35-mm culture dishes with glass coverslip bottoms (MatTek, Ashland, MA). Experiments were performed with FDB fibers 1–6 h following dissociation.

### Assessment of SR Ca^2+^ store content

Freshly dissociated FDB fibers were washed with Ca^2+^/Mg^2+^-free Ringer’s solution (in mM: 146 NaCl, 5 KCl, 10 HEPES, and 11 glucose, pH 7.4, with NaOH) twice and loaded with 5 μM Fluo 3-AM (Invitrogen) dissolved in Rodent Ringer’s solution for 20–30 min. Fibers were then washed three times in Rodent Ringer’s solution with gentle agitation. Fluo 3-AM-loaded fibers bathed in Rodent Ringer’s solution were then placed on the stage of an LSM META scanning laser confocal microscope (Carl Zeiss, Inc., Jena, Germany) and viewed under ×10 magnification. *N*-benzyl-*p*-toluenesulfonamide (BTS; 100 μM; Sigma-Aldrich) was always present in the bath solution (∼25 °C) to prevent contractions. The Fluo-3 dye was excited with the 488 nm line of an argon laser (30 mW maximum output, operated at 50 % or 6.3 A, attenuated to 1–2 %). The emitted fluorescence was directed to a photomultiplier equipped with a 505-nm long-pass filter. Confocal fluorescence intensity data were digitized at 8 bits, with the photomultiplier gain and offset adjusted such that maximum pixel intensities were no more than ∼70 % saturated and cell-free areas had close to zero intensity. Application of 4-chloro-*m*-cresol (4-C*m*C; Pfaltz and Bauer, Waterbury, CT) via a manually operated, gravity-driven global perfusion system was used to deplete the SR of Ca^2+^. Fluorescence amplitude data are expressed as ΔF/F, where F represents the baseline fluorescence before the application of 4-C*m*C, and ΔF represents the change in fluorescence during the application of 4-C*m*C.

### Electrophysiology and whole-cell recordings of myoplasmic Ca^2+^ transients

Borosilicate patch pipettes had resistances of ≤1.0 MΩ when filled with internal solution, which consisted of (mM): 140 Cs-aspartate, 10 Cs_2_-EGTA, 5 MgCl_2_, and 10 HEPES, pH 7.4 with CsOH. FDB fibers that were selected for voltage-clamp experiments were glossy, had rounded ends, and cross-striations. Fibers meeting our visual criteria were dialyzed in the whole-cell configuration for >20 min prior to recording. To record changes in intracellular Ca^2+^ from FDB fibers, the pentapotassium salt form of Fluo 3 single wavelength Ca^2+^ indicator dye (Invitrogen) was added to the standard internal solution (see above) for a final concentration of 200 μM. The external solution contained (mM): 145 TEA-methanesulfonic acid, 10 CaCl_2_, 10 HEPES, 2 MgCl_2_, 1 4-aminopyridine, 0.1 anthracene-9-carboxylic acid, 0.002 tetrodotoxin, pH 7.4 with TEA-OH. Ten micromolar BTS was added to the standard external solution for all whole-cell experiments. Following the establishment of the whole-cell configuration, the dye was allowed to diffuse into the cell interior for no less than 20 min. A 100 W mercury illuminator and a set of fluorescein filters were used to excite the dye present in the voltage-clamped fiber. A computer-controlled shutter was used to block illumination in the intervals between test potentials. Fluorescence emission was measured by means of a fluorometer apparatus (Biomedical Instrumentation Group, University of Pennsylvania). Fluorescence data are expressed as ΔF/F, where ΔF represents the change in peak fluorescence from baseline during the test pulse and *F* is the fluorescence immediately prior to the test pulse minus the average background fluorescence. The peak value of the fluorescence change (ΔF/F) for each test potential (*V*) was fitted according to:

1 $$ \left(\varDelta \mathrm{F}/F\right) = {\left(\varDelta \mathrm{F}/F\right)}_{\max }/\left\{1+ \exp \left[\left({V}_F-V\right)/{k}_F\right]\right\}, $$

where (ΔF/F)_max_ is the maximal fluorescence change, *V*_*F*_ is the potential causing half the maximal change in fluorescence, and *k*_*F*_ is a slope parameter.

All electrophysiological data were acquired with an Axon 200B patch-clamp amplifier and a CV 203BU headstage (both Molecular Devices, Sunnyvale, CA). L-type Ca^2+^ currents were recorded with the same external solution used to record myoplasmic Ca^2+^ transients described above. A solution containing (in mM): 145 TEA-methanesulfonic acid, 10 CaCl_2_, 10 HEPES, 2 MgSO_4_, 1 4-aminopyridine, 0.1 anthracene-9-carboxylic acid, 0.002 tetrodotoxin, 1 LaCl_3_, 0.5 CdCl_2_, pH 7.4 with TEA-OH were used to record intramembrane gating charge movements. Linear components of leak and capacitive current were corrected with −P/4 online subtraction protocols. Output filtering was at 2–5 kHz and digitization was either at 5 kHz (currents) or 10 kHz (charge movements). Cell capacitance (*C*_*m*_) was determined by integration of a transient from −80 to −70 mV using Clampex 10.3 (Molecular Devices) and was used to normalize charge movement (nC/μF) and current amplitude (pA/pF). The average value of *C*_*m*_ for all fibers used in the study was 2.4 ± 0.1 nF (*n* = 160 fibers; please see Additional file 1: Figure S1 for *C*_*m*_ data for each experimental group). To minimize voltage error, the time constant for the decay of the whole-cell capacity transient was reduced as much as possible using the analog compensation circuit of the amplifier; the average values of *τ*_*m*_ and *R*_*a*_ were 1.0 ± 0.0 ms and 456 ± 21 kΩ, respectively. Current-voltage (I-V) curves were fitted according to:

2 $$ I = {G}_{\max }*\ \left(V - {V}_{\mathrm{rev}}\right)\ /\ \left\{1 + \exp \left[-\left(V - {V}_{1/2}\right)\ /\ {k}_G\right]\right\}, $$

where *I* is the normalized current for the test potential *V*, *V*_rev_ is the reversal potential, *G*_max_ is the maximum channel conductance, *V*_1/2_ is the half-maximal activation potential and *k*_*G*_ is the slope factor. For charge movements, the initial non-linear outward transient “Q_ON_” was normalized to *C*_*m*_ and plotted as a function of test potential (*V*) and the resultant Q_ON_ − V relationships were fitted according to:

3 $$ {\mathrm{Q}}_{\mathrm{ON}} = {\mathrm{Q}}_{\max }/\left\{1+ \exp \left[\left({V}_Q-V\right)/{k}_Q\right]\right\}, $$

where Q_max_ is the maximal Q_ON_, *V*_*Q*_ is the potential causing movement of half the maximal charge, and *k*_*Q*_ is a slope parameter. All electrophysiological and Ca^2+^ imaging experiments were performed at room temperature (~25 °C).

### Immunoblotting

The hindlimb muscles from wild-type and SOD1^G93A^ mice (tibialis anterior, gastrocnemius, and FDB) were promptly removed following sacrifice, flash-frozen in liquid nitrogen, and stored at −80 °C for later use. At a later time, muscle tissue was homogenized by centrifugation with aluminum beads (NextAdvance, Averill Park, NY) in lysis buffer (50 mM Tris, 1 % SDS, Bio-Rad, Hercules, CA). Sample protein concentration was determined using the Bradley method (BCA assay kit, ThermoFisher, Pittsburgh, PA); lysates were divided into 30–50 μg aliquots. After addition of Laemmli buffer (Bio-Rad), proteins were separated by standard SDS-PAGE (10 % gels; Bio-Rad), transferred to nitrocellulose and non-specific immunoreactivity was blocked with 5 % non-fat dry milk (Kroger, Cincinnati, OH). Primary antibodies used for immunoblotting were mouse anti-Ca_V_1.1 (also referred to as mAB 1A; 1:1000; ThermoScientific, Rockford, IL), mouse α-actin (1:1000; Sigma-Aldrich), and rabbit histone H3 (1:1000; Cell Signaling, Danvers, MA). Goat anti-mouse and anti-rabbit HRP-conjugated secondary antibodies were obtained from Southern Biotech (1:10000; Birmingham, AL). Blots were visualized with SuperSignal West Pico kit (ThermoFisher) viewed on a FluorChem HD2 scanner (Alpha Innotech, San Leandro, CA). Blots were stripped using Restore Western Blot Stripping Buffer (ThermoScientific). Cellular Ca_V_1.1 protein levels measured using ImageJ densitometry (National Institutes of Health, Bethesda, MD) and were normalized to α-actin expression in the same sample (i.e., lane).

### Analysis

All data are presented as mean ± SEM. Statistical comparisons were made by two-tailed, unpaired *t* test with *P* < 0.05 considered significant. Figures were made using the software program SigmaPlot (version 11.0, SSPS Inc., San Jose, CA).

## Results and discussion

### EC coupling is impaired in symptomatic SOD1^G93A^ FDB fibers

**Fig. 1:**
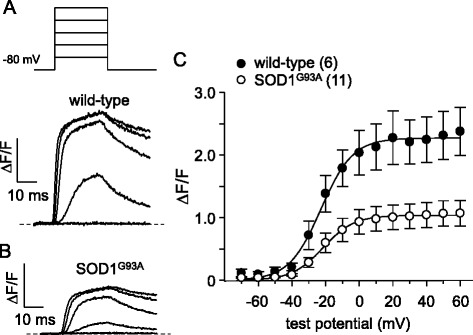
EC coupling is impaired in symptomatic global SOD1^G93A^ FDB fibers. Representative voltage-induced myoplasmic Ca^2+^ transients elicited by 25 ms depolarizations from −80 to −60, −40, −20, 0, and 20 mV from FDB fibers harvested from a wild-type C57BL/6J mouse (**a**) and a symptomatic SOD1^G93A^ mouse (**b**). The peak ΔF/F-V relationships for wild-type and symptomatic SOD ^G93A^ fibers are presented in panel (**c**). Ca^2+^ transients were evoked at 0.1 Hz by test potentials ranging from −70 mV through +60 mV in 10 mV increments. The *smooth curves* are plotted according to Eq. 1 with the respective fit parameters shown in Table 1. The numbers of analyzed fibers are indicated in *parentheses*. Throughout, *error bars* represent ± SEM

Like humans afflicted with ALS, mice engineered to carry ALS-linked mutations in SOD1 display substantial muscle atrophy and decreased specific force [30, 32]. Declines in specific force that resemble that occurring in ALS [30] have previously been attributed to reduced membrane expression of Ca_V_1.1 [33–35] and experimental knockdown of Ca_V_1.1 expression causes marked atrophy [36]. Taken together, these observations raise the question of whether the primary function of Ca_V_1.1—voltage-sensor for EC coupling—is impaired in skeletal muscle of a mutant SOD1 ALS model. To address this question, we dialyzed flexor digitorum brevis (FDB) fibers isolated from congenic SOD1^G93A^ mice and age-matched C57BL/6J wild-type control mice with cell-impermeant Fluo 3 Ca^2+^ indicator dye and recorded depolarization-induced myoplasmic Ca^2+^ transients in the whole-cell configuration (cf. ref. [37]). As shown in Fig. 1a, we observed robust SR Ca^2+^ release in response to step depolarizations in Fluo 3-loaded wild-type control fibers (ΔF/*F* = 2.3 ± 0.4, *n* = 6). By contrast, depolarization-induced SR Ca^2+^ release was substantially decreased in symptomatic SOD1^G93A^ fibers (ΔF/*F* = 1.0 ± 0.1, *n* = 9; *P* < 0.01; Fig. 1b, c; Table 1); no change in the voltage-dependence of transient activation was apparent (Table 1).

**Table 1:**
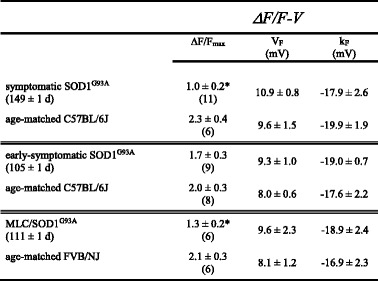
Ca^2+^ transient fit parameters

Data are given as mean ± SEM, with the numbers in parentheses indicating the number of fibers tested. Data were fit by Eq. 1. The triple thin lines separate three distinct experimental groups: (1) symptomatic, (2) early-symptomatic, and (3) MLC/SOD1^G93A^. Significant differences within each group (i.e., between a group of SOD1^G93A^ -expressing fibers and the corresponding age-matched background control fibers) are indicated (* denotes *P* < 0.05; unpaired *t* test

**Fig. 2:**
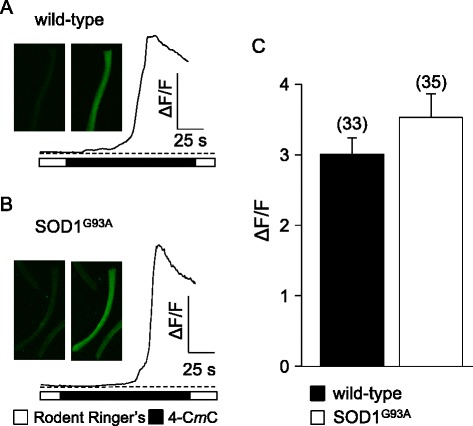
SR Ca^2+^ store content is similar in wild-type and symptomatic SOD1^G93A^ FDB fibers. Representative records of 4-C*m*C (1 mM)-induced SR Ca^2+^ release in dissociated FDB fibers harvested from a wild-type mouse (**a**) and from a symptomatic SOD1^G93A^ mouse (**b**). *Insets* show images of Fluo 3-loaded fibers before 4-C*m*C application (*left*) and at the peak of fluorescence (*right*). **c** Data summary

To determine whether the ~55 % reduction in Ca^2+^ transient amplitude in SOD1^G93A^ fibers was a consequence of a depleted SR Ca^2+^ store, we measured changes in myoplasmic Ca^2+^ levels in response to application of the RyR agonist 4-chloro-*m*-cresol (4-C*m*C; 1 mM). In these experiments (Fig. 2a–c), we observed no apparent differences in the response to 4-C*m*C between symptomatic SOD1^G93A^ and age-matched wild-type fibers (ΔF/*F* = 3.5 ± 0.3, *n* = 35, and 3.0 ± 0.2, *n* = 33, respectively; *P* > 0.05).

### L-type currents and intramembrane charge movement are reduced in symptomatic SOD1^G93A FDB fibers^

**Fig. 3:**
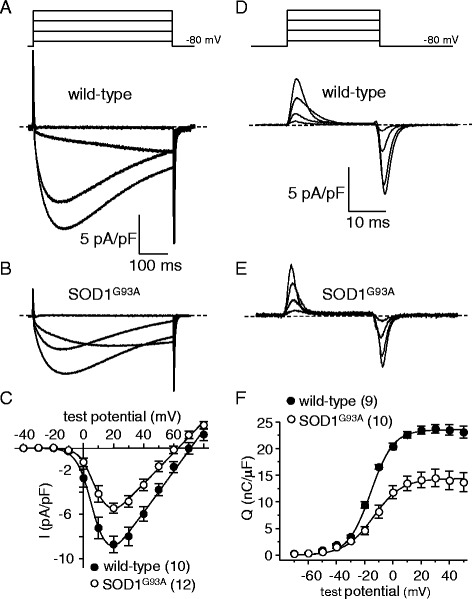
L-type currents and charge movements are reduced in symptomatic SOD1^G93A^ FDB fibers. Representative whole-cell recordings of L-type currents elicited by 500 ms step depolarizations from −80 to −20, 0, 20, and 40 mV are shown for a wild-type FDB fiber (**a**) and a symptomatic SOD1^G93A^ fiber (**b**). **c** Peak I-V relationships corresponding to the current families shown in panels (**a**) and (**b**). Currents were evoked at 0.1 Hz by test potentials ranging from −40 mV through +80 mV in 10 mV increments. Representative recordings of intramembrane charge movements elicited by 25 ms depolarizations from −80 to −40, −20, 0, and 20 mV are shown for a wild-type (**d**) and a symptomatic SOD1^G93A^ fiber (**e**). **f** Q-V relationships corresponding to the charge movements shown in panels (**d**) and (**e**). Charge movements were evoked at 0.1 Hz by test potentials ranging from −70 mV through +50 mV in 10 mV increments. The *smooth curves* in panels (**c**) and (**f**) are plotted according to Eqs. 2 and 3, respectively, with fit parameters displayed in Table 2

The impairment of EC coupling (Fig. 1) without appreciable SR Ca^2+^ store depletion (Fig. 2) suggests an alteration in Ca_V_1.1 expression and/or function. To investigate this possibility, we recorded L-type Ca^2+^ currents and intramembrane charge movements from SOD1^G93A^ fibers and wild-type fibers. Representative L-type current families from wild-type and symptomatic SOD1^G93A^ fibers are shown in Fig. 3a, b, respectively. We observed a ~40 % reduction in the peak current density in symptomatic SOD1^G93A^ fibers relative to that observed in age-matched wild-type fibers (*I*_dens_ = −5.4 ± 0.5 pA/pF, *n* = 12 vs. −8.7 ± 0.8 pA/pF, *n* = 10, respectively, at +20 mV; *P* < 0.001; Fig. 3c). In addition, we observed a similar (~40 %) reduction in maximal charge movement for SOD1^G93A^ fibers compared to wild-type fibers (*Q*_max_ = 14.1 ± 1.4 nC/μF, *n* = 10 vs. 23.4 ± 0.8 C/μF, *n* = 9, respectively; *P* < 0.005; Fig. 3d–f).

**Fig. 4:**
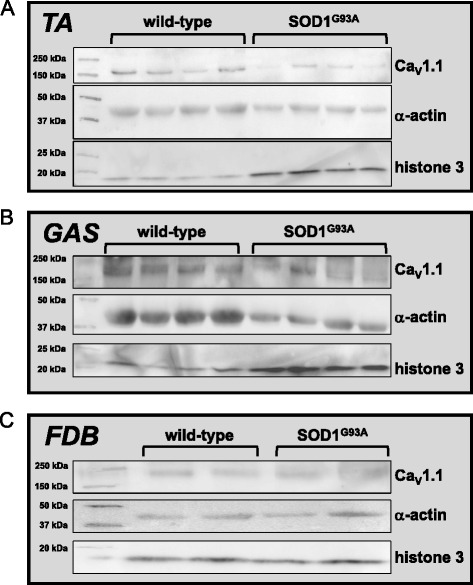
Western blot analysis of Ca_V_1.1 protein levels in symptomatic SOD1^G93A^ skeletal muscle. **a** western blots of lysates obtained from four different tibialis anterior (**b**) gastrocnemius muscles of age-matched wild-type and symptomatic SOD1^G93A^ mice are shown (*N* = 4 for both groups). In each case, the blots were probed with a monoclonal antibody directed to Ca_V_1.1 (*top* panels), then stripped and reprobed sequentially for α-actin (*middle* panels) and then histone H3 (*bottom* panels). A protein ladder standard resides in lane 1 of each blot; molecular weights are indicated in kDa. **c** A western blot of lysates obtained from FDB muscles of age-matched wild-type and symptomatic SOD1^G93A^ mice are shown (*N* = 2 for both groups). The blot was first probed with a monoclonal antibody directed to Ca_V_1.1 (*top* panel) and then stripped and reprobed sequentially with antibodies directed to α-actin (*middle* panel) and then histone H3 (*bottom* panel). For each blot, changes in expression were determined by comparing the intensity of each Ca_V_1.1 band in the *top* panel to the corresponding actin band the *middle* panel. For each panel, similar results were observed in at least three experiments

We also measured protein levels of Ca_V_1.1 in the tibialis anterior, gastrocnemius, and FDB muscles via western blot. As shown in Fig. 4a—top, we observed a clear reduction in Ca_V_1.1 levels in tibialis anterior preparations (band intensity relative to α-actin = 0.40 ± 0.03 for wild-type and 0.16 ± 0.02 for symptomatic; *P* < 0.001; both *N* = 4; Fig. 4a—top panel) that reflected our electrophysiological measurements (Fig. 3). For gastrocnemius, total Ca_V_1.1 expression was only marginally insignificant (relative band intensity = 0.32 ± 0.04 and 0.20 ± 0.04 for wild-type and symptomatic, respectively; *P* = 0.074; *N* = 4: Fig. 4b—top panel). A reduction in Ca_V_1.1 plasma membrane expression in FDB fibers was not detectable by western blot (relative band intensity = 0.54 ± 0.09 and 0.62 ± 0.12 for wild-type and symptomatic, respectively; Fig. 4c—top panel). It is important to note here that quantification of these biochemical data was extremely difficult because the expression level of all reference gene products used in our experiments also changed. α-actin was found to be the most stable marker, and we used it to calculate changes in Ca_V_1.1 band intensity (Fig. 4a–c—middle panels). Still, α-actin is not an ideal reference as it does fluctuate somewhat between wild-type and symptomatic SOD1^G93A^ muscle. The levels of histone 3 were greatly and evenly increased in the symptomatic SOD1^G93A^ tibilias anterior and gastrocnemius muscles (Fig. 4a, b—bottom panels) and unchanged in FDB (Fig. 4c—bottom panel) preparations. These experiments further supported the idea that any observed downregulation of Ca_V_1.1 was not a consequence of uneven gel loading. Likewise, Coomassie staining of the same samples used in western blots was similar between wild-type and SOD1^G93A^ muscle (Additional file 2: Figure S2A–C).

### Impaired Ca_V_1.1 function in early-symptomatic SOD1^G93A^ FDB fibers

**Fig. 5:**
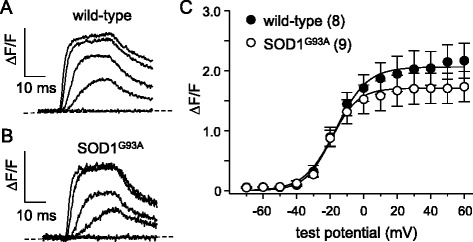
EC coupling is not significantly impaired in early-symptomatic SOD1^G93A^ FDB fibers. Representative Ca^2+^ transients elicited by 25 ms depolarizations from −80 to −60, −40, −20, 0, and 20 mV from fibers harvested from a wild-type mouse (**a**) and from an early-symptomatic SOD1^G93A^ mouse (**b**). ΔF/*F*-V relationships are shown in (**c**)

**Fig. 6:**
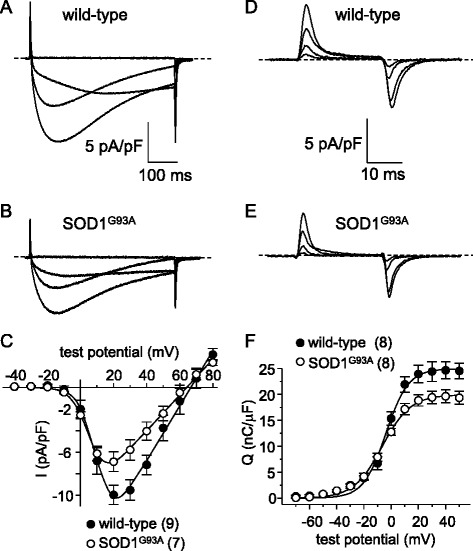
L-type currents and charge movements are reduced in early-symptomatic SOD1^G93A^ FDB fibers. Representative L-type currents elicited by 500 ms depolarizations from −80 to −20, 0, 20, and 40 mV are shown for a wild-type FDB fiber (**a**) and an early-symptomatic SOD1^G93A^ fiber (**b**). **c** Peak I-V relationships corresponding to the current families are shown in panels (**a**) and (**b**). Representative charge movement recordings elicited by 25 ms depolarizations from −80 to −40, −20, 0, 20 mV are shown for a wild-type (**d**) and a pre-symptomatic SOD1^G93A^ fiber (**e**). **f** Q-V relationships. The *smooth curves* in panels (**c**) and (**f**) are plotted according to Eq. 2 and Eq. 3, respectively, with fit parameters displayed in Table 2

Since muscle weakness and atrophy are known to precede overt locomotor effects of SOD1^G93A^ expression (e.g., abnormal gait, tremor, etc.) [38, 39], we sought to determine whether the observed impairment of Ca_V_1.1 activity in SOD1^G93A^ mouse muscle began in the early stages of presentation. We first examined EC coupling in FDB fibers of early-symptomatic SOD1^G93A^ mice and age-matched wild-type mice. In this case, we observed no significant change in Ca^2+^ transient amplitude for early-symptomatic SOD1^G93A^ fibers relative to wild-type fibers (2.0 ± 0.3 ΔF/*F*, *n* = 8 vs. 1.7 ± 0.3 ΔF/*F*, *n* = 9, respectively; *P* > 0.05 Fig. 5a–c). Despite the lack of significant effect on Ca^2+^ transient amplitude, we observed a ~30 % decrease in peak L-type current amplitude between early-symptomatic SOD1^G93A^ fibers and wild-type control fibers (*I*_dens_ = −6.9 ± 0.9 pA/pF, *n* = 7 vs. 10.0 ± 0.9 pA/pF, *n* = 6, respectively; *P* < 0.05; Fig. 6a–c). Charge movements were also reduced (~20 %) in early-symptomatic SOD1^G93A^ fibers relative to wild-type fibers (*Q*_max_ = 18.8 ± 1.1 nC/μF, *n* = 8 vs. 23.9 ± 1.7 nC/μF, *n* = 8, respectively; *P* < 0.05; Fig. 6d–f; Table 2).

**Table 2:**
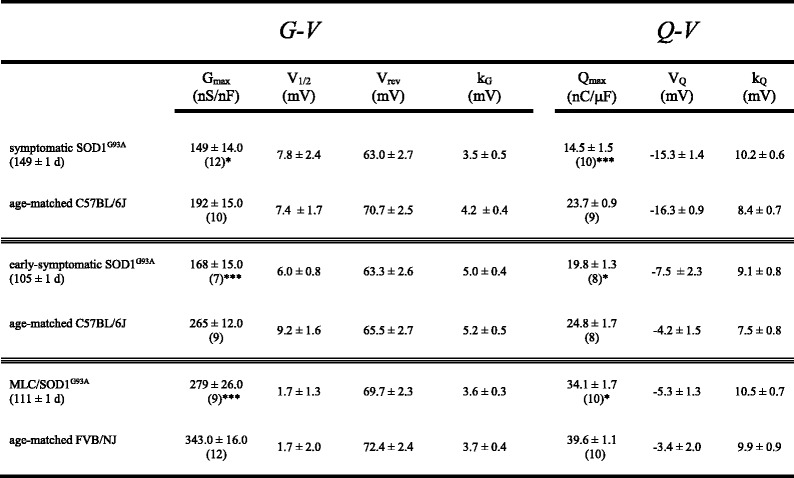
Conductance and charge movement fit parameters

Data are given as mean ± SEM, with the numbers in parentheses indicating the number of FDB fibers tested. Conductance and charge movement data were fit by Eqs. 2 and 3, respectively. As in Table 1, the triple thin lines separate three distinct experimental groups: (1) symptomatic, (2) early-symptomatic, and (3) MLC/ SOD1^G93A^. Again, significant differences within each group are indicated (* denotes *P* < 0.05; *** denotes *P* < 0.001; unpaired t test)

**Fig. 7:**
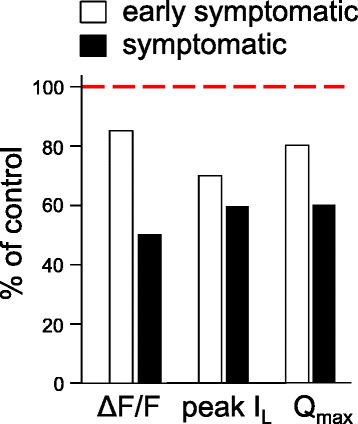
Summary of results obtained with global SOD1^G93A^ fibers. EC coupling (ΔF/*F*), L-type Ca^2+^ current (peak I_L_)_,_ and maximal charge movement (*Q*_max_) for early-symptomatic (105 ± 1 d; *N* = 6; *white bars*) and symptomatic global SOD1^G93A^ (149 ± 1 d; *N* = 9; *black bars*) fibers are represented as a percentage of corresponding values for the respective age-matched wild-type fiber groups

In Fig. 7, we summarize our results with symptomatic and early-symptomatic SOD1^G93A^ FDB fibers. There, EC coupling (ΔF/*F*), L-type channel current amplitude (peak I_L_) and intramembrane charge movement (*Q*_max_) are normalized by the corresponding age-matched wild-type control value for each parameter. For symptomatic SOD1^G93A^ fibers, the reductions in EC coupling, channel function, and charge movement are quite evident (Fig. 7—black bars). Moreover, these impairments of Ca_V_1.1 function were already developing in early-symptomatic SOD1^G93A^ mice before substantial denervation had occurred (Fig. 7—white bars), suggesting that muscle-specific events are contributing to SOD1^G93A^-dependent alterations in Ca_V_1.1 function.

### EC coupling, L-type currents, and charge movement are reduced in FDB fibers expressing SOD1^G93A^ autonomously in the skeletal muscle

Our experiments with early-symptomatic SOD1^G93A^ mice raise the possibility that the reduction of Ca_V_1.1 expression precedes overt neurological symptoms of ALS. Nonetheless, a degree of uncertainty exists concerning the contribution of denervation. On a more stringent level, the innervation profiles of the individual fibers used in our patch-clamp experiments were virtually impossible to assess accurately. To determine nerve-independent effects, we utilized a mouse model where transgenic expression of SOD1^G93A^ is limited to skeletal muscle (MLC/SOD1^G93A^) [6]. This mouse line enabled us to examine whether events occurring locally in skeletal muscle can cause impaired Ca_V_1.1 function because substantial neuromuscular junction defects in such models do not develop until at least 8 months of age ([6, 7]; A.M., unpublished results).

**Fig. 8:**
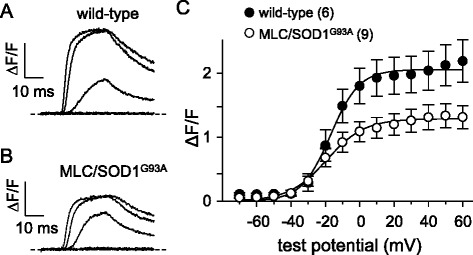
EC coupling is impaired in MLC/SOD1^G93A^ FDB fibers. Representative Ca^2+^ transients elicited by 25 ms depolarizations from −80 to −60, −40, −20, 0, and 20 mV in fibers obtained from a wild-type FVB/NJ mouse (**a**) and a MLC/SOD1^G93A^ mouse (**b**). ΔF/*F*-V relationships are shown in (**c**)

**Fig. 9:**
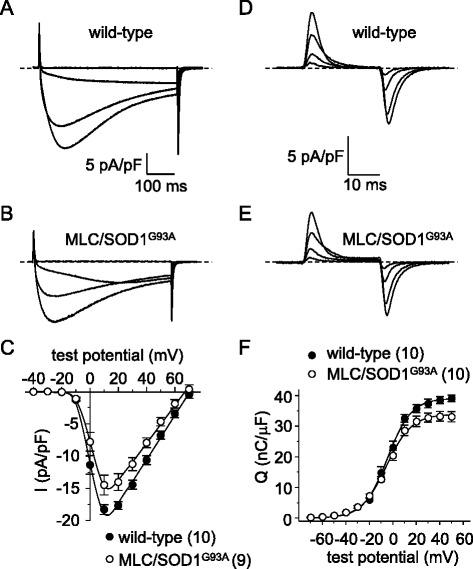
L-type currents and charge movements are reduced in MLC/SOD1^G93A^ FDB fibers. Representative L-type currents elicited by 500 ms depolarizations from −80 to −20, 0, 20, and 40 mV are shown for a wild-type FDB fiber (**a**) and an MLC/SOD1^G93A^ fiber (**b**). **c** Peak I-V relationships corresponding to the current families shown in panels (**a**) and (**b**). Representative charge movement recordings elicited by 25 ms depolarizations from −80 to −40, −20, 0, and 20 mV are shown for a wild-type (**d**) and an MLC/SOD1^G93A^ fiber (**e**). **f** Q-V relationships corresponding to the charge movements shown in panels (**d**) and (**e**). The *smooth curve*s in panels (**c**) and (**f**) are plotted according to Eqs. 2 and 3, respectively, with fit parameters displayed in Table 2

We recorded from fibers obtained from 4-month-old MLC/SOD1^G93A^ mice. In these experiments, myoplasmic Ca^2+^ transients recorded from MLC/SOD1^G93A^ fibers were significantly reduced compared to their aged-matched wild-type FVB/NJ counterparts (ΔF/*F* = 1.3 ± 0.2, *n* = 9 vs. 2.1 ± 0.3, *n* = 6, respectively; *P* < 0.05; Fig. 8a–c). As with global SOD1^G93A^ fibers (Fig. 1), the changes in Ca^2+^ transient amplitude were not attributable to compromised SR Ca^2+^ stores as no significant difference in the response to 4-C*m*C between MLC/SOD1^G93A^ and age-matched wild-type fibers was evident (ΔF/*F* = 5.0 ± 0.6, *n* = 27, and 4.4 ± 0.3, *n* = 17, respectively; *P* > 0.05). We also observed smaller (~20 %) L-type current amplitudes in MLC/SOD1^G93A^ fibers relative to wild-type fibers (*I*_dens_ = −14.5 ± 1.5 pA/pF, *n* = 9 vs. −18.4 ± 0.6 pA/pF, *n* = 12, respectively; *P* < 0.05; Fig. 9a–c). Charge movements were reduced by similar degree (~15 %) in MLC/SOD1^G93A^ fibers (*Q*_max_ = 34.1 ± 1.7 nC/μF, *n* = 10 vs. 39.6 ± 1.1 nC/μF for wild-type, *n* = 10, respectively; *P* < 0.05; Fig. 9d–f; Table 2). Taken together, our results with MLC/SOD1^G93A^ fibers indicate that muscle-specific expression of SOD1^G93A^ ultimately causes a reduction in the total number of functional Ca_V_1.1 channels present in the transverse tubules.

### Discussion

In this study, we found that FDB fibers harvested from symptomatic global SOD1^G93A^ mice had substantially reduced EC coupling relative to fibers of age-matched wild-type mice (Fig. 1). The impairment of EC coupling was not a consequence of a depleted SR Ca^2+^ store (Fig. 2), but appeared to be the result of reduced L-type Ca^2+^ channel membrane expression (though we cannot discount the presence of electrically silent channels). In particular, L-type Ca^2+^ current (Fig. 3a–c) and intramembrane charge movement (Fig. 3d–f) were both decreased by ~40 % in symptomatic SOD1^G93A^ FDB fibers. The observed reductions in Ca_V_1.1 function in symptomatic SOD1^G93A^ fibers resembled the effect of application of ALS patient serum to normal rat extensor digitorum longus muscle [26, 27], but our results imply that these impairments develop more slowly over time.

Our data indicating that EC coupling and L-type current were compromised in symptomatic SOD1^G93A^ muscle fibers contrast with those of another group who found that depolarization-dependent Ca^2+^ transients were slightly augmented in SOD1^G93A^ mice (B6SJL-Tg(SOD1*G93A)1Gur/J) as a consequence of insufficient local mitochondrial Ca^2+^ buffering near the neuromuscular junction [40, 41]. Our findings also differ with those of another study which also examined Ca^2+^ handling in FDB fibers obtained from symptomatic SOD1^G93A^ mice (B6.Cg-Tg(SOD1*G93A)1Gur/J). In this latter study [42], the authors observed virtually no difference in the amplitude of Ca^2+^ transients evoked by maximal field stimulation frequencies (100 Hz) using ratiometric Ca^2+^-sensitive dyes. It was concluded that the ability of Ca_V_1.1 to engage EC coupling was normal in symptomatic SOD1^G93A^ superficial gastrocnemius muscle because western blots showed no change in Ca_V_1.1 expression [42]. By contrast, our western blot analysis of whole-cell lysates obtained from symptomatic SOD1^G93A^ tibialis anterior muscles indicated a clear reduction in total Ca_V_1.1 expression (Fig. 4a). We did not observe significant differences for either gastrocnemius or FDB (Fig. 4b, c), though the band intensity difference in gastrocnemius was marginal (*P* = 0.074). The ambiguous nature of our biochemical results underscores the critical importance of our voltage-clamp approach to determine the relative number of functional Ca_V_1.1 channels in the plasma membrane.

One reasonable explanation for the observed decreases in Ca^2+^ transient amplitude, peak L-type current density, and maximal charge movement is that SOD1^G93A^-expressing muscle undergoes fast-twitch (type IIb or IIx) to slow or intermediate (type I or type Ia, respectively) fiber-type switching [6, 30]. In an earlier study, slow-twitch rat soleus fibers were found to support voltage-dependent SR Ca^2+^ release only about half as well as fast-twitch fibers dissected from EDL of the same animals [43, 44]. Likewise, radioactive 1,4-dihydropyridine binding of the soleus was reduced ~50 % compared to that of EDL in the same study. In view of this difference in Ca_V_1.1 expression between general fiber types, it is possible that we recorded from a population more heavily weighted in type I or type IIa fibers and that our data reflect fiber-type switching. Nevertheless, the dramatic alteration in the expression profile of Ca_V_1.1 that accompanies fiber-type switching appears to be a downstream consequence of mutant SOD1 expression. Future experiments with more complex models that enable the distinction amongst fiber types in live cells are required to investigate this idea further.

Importantly, we also found that the impairment of Ca_V_1.1 function in SOD1^G93A^ mice began to develop prior the presentation of classical ALS symptoms. Though the reduction in depolarization-induced Ca^2+^ transients were not significantly reduced in early-symptomatic SOD1^G93A^ muscle fibers (Fig. 5), decreased L-type current amplitude and intramembrane charge movement were clearly evident (Fig. 6). A number of explanations could account for this apparently discordant result. For example, the early-symptomatic SOD1^G93A^ mice may have had a dissimilar RyR (type 1 or type 3) complement than the symptomatic mice or there have been a compensatory reversion to a juvenile RyR1 splice variant (ASI+) that supports greater SR Ca^2+^ release in response to depolarization [45, 46]. In any event, the results of our experiments with early-symptomatic SOD1^G93A^ fibers suggested that the impairment of Ca_V_1.1 function manifested in symptomatic SOD1^G93A^ fibers arises, at least in part, from signals that originate in muscle (Figs. 5, 6, and 7). To test this idea, we assessed Ca_V_1.1 function in fibers obtained from 4-month-old MLC/SOD1^G93A^ mice in which expression of the mutant SOD1 protein is restricted to skeletal muscle. In these experiments, we observed ~35, ~20, and ~15 % reductions in Ca^2+^ transient amplitude, peak L-type current density, and maximal charge movement, respectively (Figs. 8 and 9). The reductions in L-type current amplitude and charge movement resembled the reductions in the parameters observed in early-symptomatic global SOD1^G93A^ fibers. Interestingly, the impact of muscle-specific expression of SOD1^G93A^ on EC coupling was greater than that observed in early-symptomatic global fibers. Previous ultrastructural analysis of the MLC/SOD1^G93A^ strain suggests that concurrent uncoupling of triad junctions with transverse tubules may amplify the EC coupling impairment [6]. Together, the ultrastructural and electrophysiological changes induced by muscle-specific expression of SOD1^G93A^ can reasonably account for marked reductions in specific force observed in both fast- and slow-twitch hindlimb muscles of MLC/SOD1^G93A^ mice [6]. Thus, the results of our experiments with MLC/SOD1^G93A^ fibers support the idea that muscle-specific events also make a significant contribution to ALS pathology, similar to what has been observed previously with myotonic dystrophy [47] and Huntington’s disease [48].

We did not observe a decrease in total membrane capacitance in SOD1^G93A^-expressing fibers, indicating that there was not substantial atrophy and/or loss of tubular surface area in the fibers that we examined electrophysiologically (Additional file 1: Figure S1). Based on the indisputable fact that atrophy is characteristic of both global and muscle-targeted SOD1^G93A^ muscles, we think that our data are representative of a subpopulation of fibers healthy enough to survive the dissociation process and to meet the set visual criteria for voltage-clamp experiments (see Methods). If this is indeed the case, the reductions in voltage-dependent SR Ca^2+^ release, charge movement, and L-type current in the entire population of SOD1^G93A^ fibers are likely more severe (and earlier onset) than we report.

Until recently, the involvement of Ca_V_1.1 in the maintenance of muscle mass and composition was impossible to investigate because mice null for Ca_V_1.1 (dysgenic) die immediately following parturition [49]. To circumvent the perinatal lethality of Ca_V_1.1 deletion, Piétri-Rouxel and colleagues delivered anti-Ca_V_1.1 siRNA to the mouse hindlimb muscle with a serotype 1 Adeno-Associated Virus (AAV1) vector [36]. Using this approach, they demonstrated that targeted, long-term suppression of L-type channel expression via induced exon-skipping produced prominent atrophy and extensive fibrosis. This atrophic effect of experimental downregulation of Ca_V_1.1 expression suggested that pathological decreases in L-type channel activity, such as those we have observed in the two SOD1^G93A^ models utilized in this study, may also lead to muscle degeneration and, possibly, contribute to the destabilization of neuromuscular junctions early in the progression of ALS. Our current work provides initial support for this idea, but further work is needed to reveal the precise mechanism. The first step will be to determine whether the downregulation of the channel and/or its auxiliary subunits occurs at the message level or post-translationally. For the former, quantification of Ca_V_1.1 subunit message levels by qRT-PCR should be revealing. In regard to the latter, the chronic upregulation of expression the RGK family small GTP binding protein Rad (*R*as-like *A*ssociated with *D*iabetes) — a potent constitutively active inhibitor of Ca_V_1.1 [50, 51]—has been demonstrated in muscle of sporadic ALS patients of unknown aetiology and two established familial ALS mouse models (SOD1^G93A^ and SOD1^G86R^) [52, 53]. The increases in muscle Rad expression were attributed to elevated cellular oxidative stress levels and coincided with the onset of spinal motoneuron death [53]. Without being overspeculative, these observations raise the possibility that such chronic enhancement of muscle Rad expression may contribute to both atrophy and the dissolution of neuromuscular junctions in human ALS [53].

## Conclusions

We report that myoplasmic Ca^2+^ transient amplitude, L-type current density, and intramembrane charge movement are progressively downregulated in flexor digitorum brevis (FDB) fibers obtained from SOD1^G93A^ mice, the most extensively utilized mouse model of familial ALS. We also observed impairments of EC coupling, L-type current density, and charge movement in fibers of mice expressing SOD1^G93A^ only in the skeletal muscle, signaling a muscle-specific contribution to SOD1^G93A^-induced downregulation of Ca_V_1.1 expression and/or function. Since Ca_V_1.1 is a positive regulator of muscle mass, our results suggest that altered Ca_V_1.1 activity is a contributor to the muscle remodeling that occurs in ALS patients prior to the presentation of overt neurological symptoms.

## Abbreviations

4-C*m*C, 4-chloro-*m*-cresol; AAV1, serotype 1 adeno-associated virus; ALS, amyotrophic lateral sclerosis; EC, excitation-contraction; EDL, extensor digitorum longus; FDB, flexor digitorum brevis; GAPDH, glyceraldehyde-3-phosphate dehydrogenase; IGF-1, insulin-like growth factor 1; RyR, ryanodine-sensitive intracellular Ca^2+^ release channel; SOD1, Cu/Zn superoxide dismutase 1; SR, sarcoplasmic reticulum; TA, tibialis anterior

## Additional files

Additional file 1: Figure S1. Capacitance measurements. White bars represent the average membrane capacitance (*C*_*m*_) recorded from symptomatic SOD1^G93A^, early-symptomatic SOD1^G93A^, and MLC/SOD1^G93A^ fibers. Black bars represent the average membrane capacitance recorded from the appropriate wild-type control group (see “Methods” section). A significant difference between wild-type FVB/NJ and MLC/SOD1^G93A^ fibers is indicated (*** denotes *P* < 0.001; unpaired *t* test). (PPTX 56.7 kb) Additional file 2: Figure S2. >Coomassie staining. Coomassie staining of gels containing the same samples used in the immunoblotting experiments shown in Fig. 4: (A) tibialis anterior, (B) gastrocnemius, and (C) FDB. (PPT 109 kb)
