# Supplementary material for: Progressive impairment of CaV1.1 function in the skeletal muscle of mice expressing a mutant type 1 Cu/Zn superoxide dismutase (G93A) linked to amyotrophic lateral sclerosis
Source: Skelet Muscle. 2016 Jun 23;6:24. doi: 10.1186/s13395-016-0094-6 (PMC4918102; doi:10.1186/s13395-016-0094-6)
Supplement: Additional file 1: Figure S1. — Capacitance measurements. White bars represent the average membrane capacitance (C m) recorded from symptomatic SOD1G93A, early-symptomatic SOD1G93A, and MLC/SOD1G93A fibers. Black bars represent the average membrane capacitance recorded from the appropriate wild-type control group (see “Methods” section). A significant difference between wild-type FVB/NJ and MLC/SOD1G93A fibers is indicated (*** denotes P < 0.001; unpaired t test). (PPTX 56.7 kb) [file 13395_2016_94_MOESM1_ESM.pptx]

## Slide 1
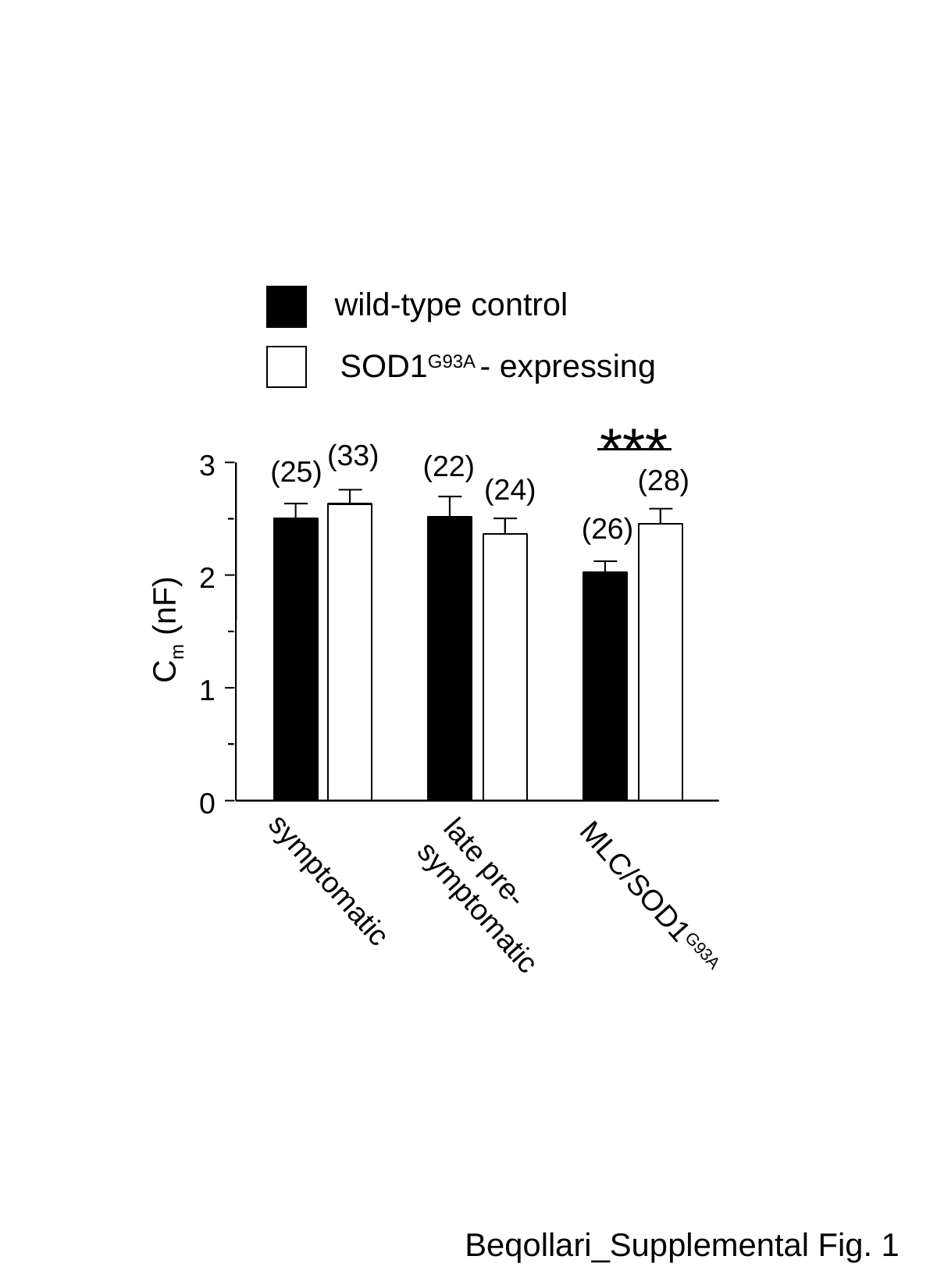

wild-type control
SOD1G93A - expressing
***
 (33)
 (25)
 (22)
 (24)
3
2
1
0
 (28)
 (26)
Cm (nF)
late pre-
symptomatic
symptomatic
MLC/SOD1G93A
Beqollari_Supplemental Fig. 1
