# Supplementary figures and images for: Progressive impairment of CaV1.1 function in the skeletal muscle of mice expressing a mutant type 1 Cu/Zn superoxide dismutase (G93A) linked to amyotrophic lateral sclerosis
Source: Skelet Muscle. 2016 Jun 23;6:24. doi: 10.1186/s13395-016-0094-6 (PMC4918102; doi:10.1186/s13395-016-0094-6)

## Slide 1
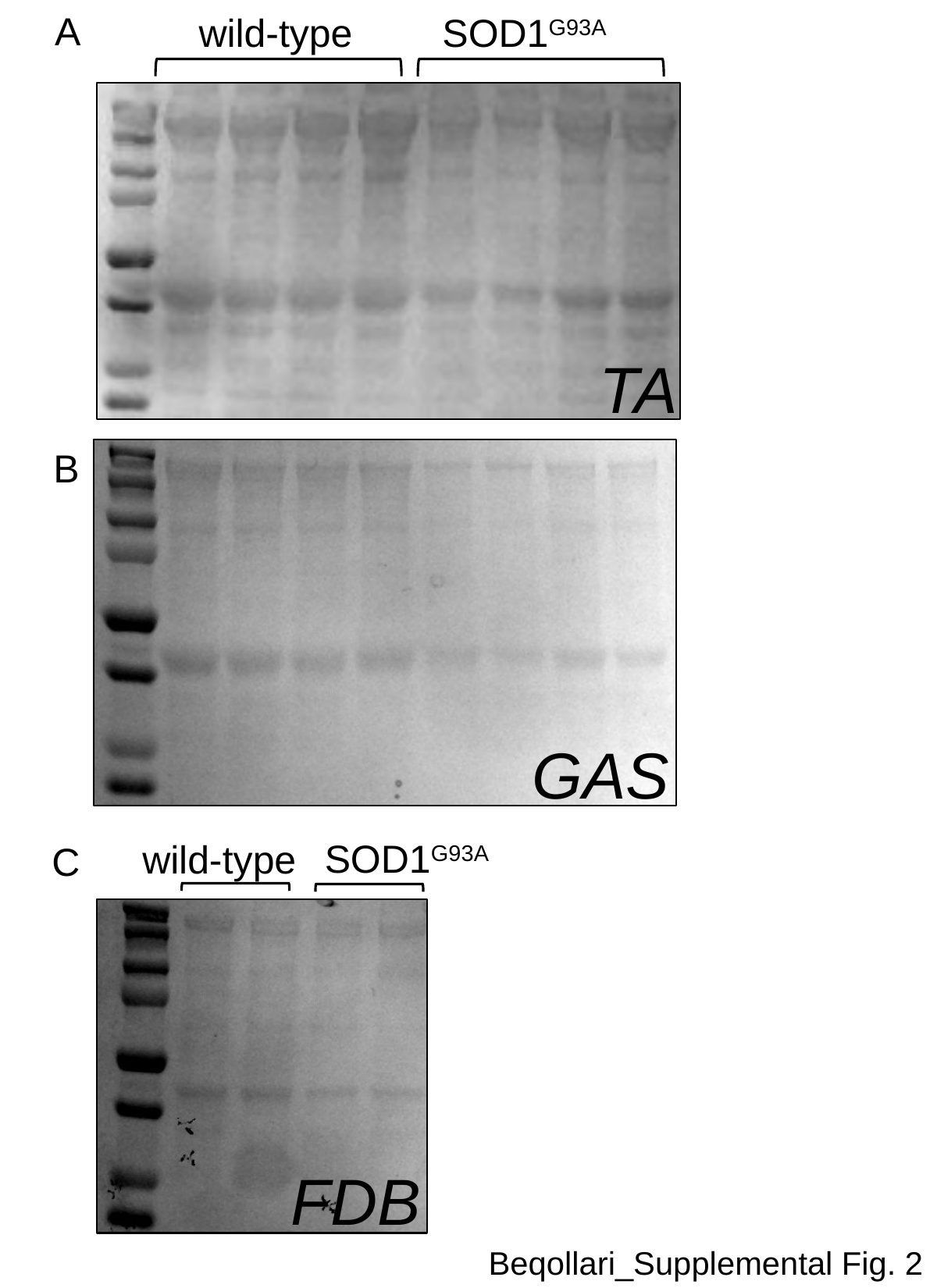

A
wild-type
SOD1G93A
TA
B
GAS
SOD1G93A
wild-type
FDB
C
Beqollari_Supplemental Fig. 2

Supplement: Additional file 2: Figure S2. — >Coomassie staining. Coomassie staining of gels containing the same samples used in the immunoblotting experiments shown in Fig. 4: (A) tibialis anterior, (B) gastrocnemius, and (C) FDB. (PPT 109 kb) [file 13395_2016_94_MOESM2_ESM.pptx]
